# Dissecting the Heterogeneity in T-Cell Mediated Inflammation in IBD

**DOI:** 10.3390/cells9010110

**Published:** 2020-01-02

**Authors:** Irma Tindemans, Maria E. Joosse, Janneke N. Samsom

**Affiliations:** Laboratory of Pediatrics, Division Gastroenterology and Nutrition, Erasmus MC-Sophia Children’s Hospital, P.O. Box 2040, 3000 CA Rotterdam, The Netherlands

**Keywords:** CD4^+^ T-cell, T helper cell, IBD, intestine

## Abstract

Infiltration of the lamina propria by inflammatory CD4^+^ T-cell populations is a key characteristic of chronic intestinal inflammation. Memory-phenotype CD4^+^ T-cell frequencies are increased in inflamed intestinal tissue of IBD patients compared to tissue of healthy controls and are associated with disease flares and a more complicated disease course. Therefore, a tightly controlled balance between regulatory and inflammatory CD4^+^ T-cell populations is crucial to prevent uncontrolled CD4^+^ T-cell responses and subsequent intestinal tissue damage. While at steady state, T-cells display mainly a regulatory phenotype, increased in Th1, Th2, Th9, Th17, and Th17.1 responses, and reduced Treg and Tr1 responses have all been suggested to play a role in IBD pathophysiology. However, it is highly unlikely that all these responses are altered in each individual patient. With the rapidly expanding plethora of therapeutic options to inhibit inflammatory T-cell responses and stimulate regulatory T-cell responses, a crucial need is emerging for a robust set of immunological assays to predict and monitor therapeutic success at an individual level. Consequently, it is crucial to differentiate dominant inflammatory and regulatory CD4^+^ T helper responses in patients and relate these to disease course and therapy response. In this review, we provide an overview of how intestinal CD4^+^ T-cell responses arise, discuss the main phenotypes of CD4^+^ T helper responses, and review how they are implicated in IBD.

## 1. Introduction

Up to 60% of lymphocytes residing in the intestinal tissue are cluster-of-differentiation 4 positive (CD4^+^) memory T-cells [1]. Upon re-exposure to antigens, CD4^+^ memory T-cells mount a rapid and highly efficient immune response. As a result, CD4^+^ memory T-cell responses are essential for protective immunity against pathogens but need to be tightly regulated to avoid inflammatory responses to harmless commensal bacteria [2]. Patients with inflammatory bowel disease (IBD) develop uncontrolled inflammatory CD4^+^ T-cell responses which can be caused by either by insufficient suppression of inflammatory CD4^+^ T-cell responses but also by insufficient host defense to pathogens, both resulting in tissue damage and chronic intestinal inflammation [3].

IBD is a heterogeneous disease and is clinically classified as Crohn’s disease (CD) or ulcerative colitis (UC) based on symptoms, disease location, and histopathological features. UC affects the colon and is a superficial ulcerative disease [4,5,6], whereas in CD inflammation can affect any part of the gastrointestinal tract, is transmural and often associated with granuloma formation [4,5,6]. A common disease denominator in all IBD patients is the infiltration of inflammatory CD4^+^ T-cells in intestinal tissue. Inflammatory T-cells steer the function of innate cells such as epithelial cells, fibroblasts, and phagocytes, thereby fueling a continuous hyperresponsiveness to microbial triggering. However, the phenotype, strength and relative contribution of the derailed inflammatory CD4^+^ T-cell response largely varies from patient to patient. The combination of the ability of T-cells to exert memory to previously encountered antigens [7], together with the persistence of commensal microbiota and a perturbed innate immune response critically determines the chronic relapsing-remitting course of the disease. Depletion of CD4^+^ T-cells is effective in treating IBD patients [8,9]. In addition, new therapeutics targeting cytokines that stimulate CD4^+^ T-cell differentiation are effective in subgroups of patients, highlighting the importance of CD4^+^ memory T-cells in driving IBD.

In this review, we describe how CD4^+^ memory T-cell responses arise, describe the functionally distinct CD4^+^ T-cell sub-populations and discuss how their function contributes to intestinal inflammation in IBD. A detailed understanding of functional CD4^+^ T-cell responses will enable stratification of IBD patients in subtypes according to their underlying immune pathology and enable identification of the most appropriate treatment for each IBD subtype.

## 2. Mechanisms of CD4^+^ T-Cell Differentiation in the Healthy Intestine

**Figure 1:**
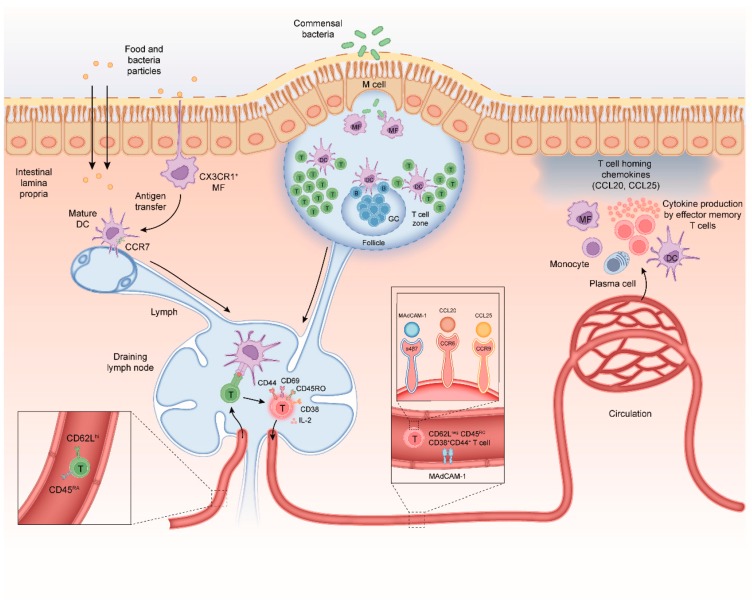
Mechanisms of CD4^+^ T-cell differentiation in the healthy intestine. (i) Antigens can be taken up by specialized microfold cells (M-cells) that can transport microbial antigens across the mucosal epithelium from the lumen to the subepithelial dome in the Peyer’s patch (PP); (ii) soluble antigens can diffuse through epithelial tight junctions and can be transferred across epithelial cells by transcellular routes and be ingested by phagocytes such as macrophages and dendritic cells (DC); and (iii) luminal antigens can also be captured by transepithelial projecting dendrites from macrophage-like CX3CR1^+^ APCs in the lamina propria. M-cells and CX3CR1^+^ APCs can transfer antigen to migratory dendritic cells that migrate from the lamina propria to inductive sites to interact with naïve CD4^+^ T-cells. Naïve CD4^+^ CD45RA chemokine receptor C-C motif receptor 7^+^ (CCR7) T-cells migrate from the peripheral blood into gut-draining lymph nodes and PP. The DC-mediated T-cell activation requires three signals; (i) T-cell receptor (TCR) stimulation with antigenic peptides in the context of major histocompatibility complex class II (MHCII), (ii) co-stimulation via CD28-CD80/CD86 engagement and (iii) cytokine signals mostly provided by APCs. Upon activation, T-cells acquire CD69, CD25 (IL-2 receptor-alpha chain) CD44 and CD45RO. T-cells down-regulate expression of CD62L, CCR7, and CD45RA and start secreting IL-2, a cytokine needed for proliferation. After a 3–4-day proliferation period the differentiated T-cells egress from the lymph node into the blood. A subpopulation of these CD45ROCD44^+^CD4^+^ T-cells will re-express CD62L^hi^ and CCR7 and provide central memory in lymphoid tissues. The remainder of the CD62L^neg^CD45ROCD44^+^CD4^+^ T-cells will migrate to the intestinal lamina propria where they reside as long-lived memory CD4^+^ T-cells. Crucially, differentiation of T-cells in the MLN is associated with imprinting of higher levels of retinoic acid-induced CD38. As this CD62L^neg^CD38^+^ effector T-cell phenotype is induced regardless of regulatory or inflammatory T-cell function it can be used to monitor gut-homing T-cells in human peripheral blood. Homing of memory-phenotype CD62L^lo^CD45ROCD44^+^CD4^+^ T-cells to the small and large intestine requires expression of the integrin α4β7 which binds to mucosal vascular addressin cell adhesion molecule 1 (MADCAM1) on endothelial cells in blood vessels. In the small intestine, epithelial cells produce the chemokine CCL25 which attracts memory T-cells expressing the receptor CCR9 Chemokine receptors involved in regulation of CD4^+^ T-cell homing to the colon at steady state are less well defined, although recently, it was discovered that G protein coupled receptor 15 (GPR15) directs CD4^+^ T-cells to the colon. During intestinal inflammation, CCR6 and its ligand CCL20 also contribute to CD4^+^ T-cell recruitment to the inflamed small and large intestine, demonstrating that CD4^+^ T-cells can use alternative homing receptors during inflammation when compared to steady state.

The intestine is continuously exposed to harmless antigens from the diet and commensal bacteria, but also provides harmful pathogens access to the body. To ensure tailored immune responses to harmless antigen and pathogens, the intestinal tissue contains a highly specialized network of innate and adaptive immune cells [10]. CD4^+^ memory T-cell responses are essential for protective immunity against pathogens but need to be tightly regulated to avoid inflammatory responses to harmless commensal bacteria [2]. Anatomically, CD4^+^ T-cells are located within both inductive and effector sites of the intestine. The mesenteric lymph nodes (MLN) and gut-associated lymphoid tissue (GALT) are the main location for priming naïve T-cell responses [10] (Figure 1). The GALT consists of the Peyer’s patches (PP) of the small intestine, the colonic patches in the colon and smaller structures referred to as solitary isolated lymphoid tissue follicles (SILT) [11]. The mucosal epithelium and underlying lamina propria harbor large populations of activated CD4^+^ memory T-cells.

Intestinal antigen presenting cells (APCs) have a key role in the initiation of adaptive immune responses to intestinal antigens. Antigens that cross the intestinal epithelium first encounter APCs in either the GALT or the lamina propria [12]. There are at least three mechanisms that contribute to antigen uptake in the intestine; (i) the follicle-associated epithelium of the Peyer’s patches contains specialized microfold cells (M-cells) that can transport microbial antigens across the mucosal epithelium from the lumen to the subepithelial dome in the Peyer’s patch [13]; (ii) soluble antigens can diffuse through epithelial tight junctions and can be transferred across epithelial cells by transcellular routes; and (iii) luminal antigens can also be captured by transepithelial projecting dendrites from macrophage-like CX3CR1^+^ APCs in the lamina propria [14,15]. M-cells and CX3CR1^+^ APCs transfer antigen to migratory dendritic cells (DCs) that migrate from the lamina propria to inductive sites in order to interact with naïve CD4^+^ T-cells [15,16,17,18,19,20] (Figure 1).

Naïve CD4^+^ T-cells migrate from the peripheral blood into gut-draining lymph nodes and Peyer’s patches using the lymphoid tissue homing receptors CD62L and chemokine receptor C-C motif receptor 7 (CCR7) [21]. T-cell activation requires three signals; (i) T-cell receptor (TCR) stimulation with antigenic peptides in the context of major histocompatibility complex class II (MHCII), (ii) co-stimulation via CD28-CD80/CD86 engagement and (iii) cytokine signals mostly provided by APCs [22,23]. Upon activation, T-cells acquire various cell surface receptors including CD69, CD25 (IL-2 receptor-alpha chain) CD44 and CD45RO. T-cells down-regulate expression of CD62L, CCR7, and CD45RA and start secreting IL-2, a key cytokine needed for proliferation. Activated T-cells proliferate and differentiate in the lymph nodes during a 3–4 days period [24] after which they egress from the lymph node into the blood. A subpopulation of these CD45ROCD44^+^CD4^+^ T-cells will re-express CD62L^hi^ and CCR7 and provide central memory in lymphoid tissues. The remainder of the CD62L^neg^CD45ROCD44^+^CD4^+^ T-cells will migrate to the intestinal lamina propria where they reside as long-lived memory CD4^+^ T-cells. Crucially, differentiation of T-cells in the MLN is associated with imprinting of higher levels of retinoic acid-induced CD38. As this CD62L^neg^CD38^+^ effector T-cell phenotype is induced regardless of regulatory or inflammatory T-cell function it can be used to monitor gut-homing T-cells in human peripheral blood [25,26].

Homing of memory-phenotype CD62L^lo^CD45ROCD44^+^CD4^+^ T-cells to the intestine is tightly regulated. In response to signals from the APC and stromal cells in the MLN the differentiating CD4^+^ T-cells up-regulate expression of the integrin α4β7 and the chemokine receptor CCR9 [27,28]. Intestinal APC-derived retinoic acid is a crucial factor promoting induction of α4β7 and CCR9 on responding CD4^+^ T-cells [29,30]. Epithelial cells in the small intestine produce the chemokine CCL25, the ligand for CCR9, and lamina propria venules express the integrin α4β7 ligand mucosal vascular addressin cell adhesion molecule 1 (MADCAM1) [31,32,33]. Hence, circulating CD4^+^ T-cells that express both α4β7 and CCR9 are recruited to the small intestinal lamina propria [34,35] (Figure 1). Expression of integrin α4β7 also contributes to accumulation of CD4^+^ T-cells in the large intestine [36,37]. Chemokine receptors involved in regulation of CD4^+^ T-cell homing to the colon at steady state are less well defined, although recently, it was discovered that G protein coupled receptor 15 (GPR15) directs CD4^+^ T-cells to the colon [38,39]. During intestinal inflammation, CCR6 and its ligand CCL20 also contribute to CD4^+^ T-cell recruitment to the inflamed small and large intestine, demonstrating that CD4^+^ T-cells can use alternative homing receptors during inflammation when compared to steady state [40]. Signals from the APC and MLN microenvironment determine the regionalization of the mucosally imprinted CD4^+^ T-cell response irrespective of the T-cell phenotype and function. Thus, both regulatory and inflammatory CD4^+^ T-cells are imprinted to home to the intestine. At steady state, the majority of CD4^+^ T-cells in lamina propria harbor a CD38^+^CD45ROCD44^+^CD62L^neg^ effector memory phenotype [25,26,41,42].

## 3. Regulatory and Inflammatory T-Cell Sub-Populations

**Figure 2:**
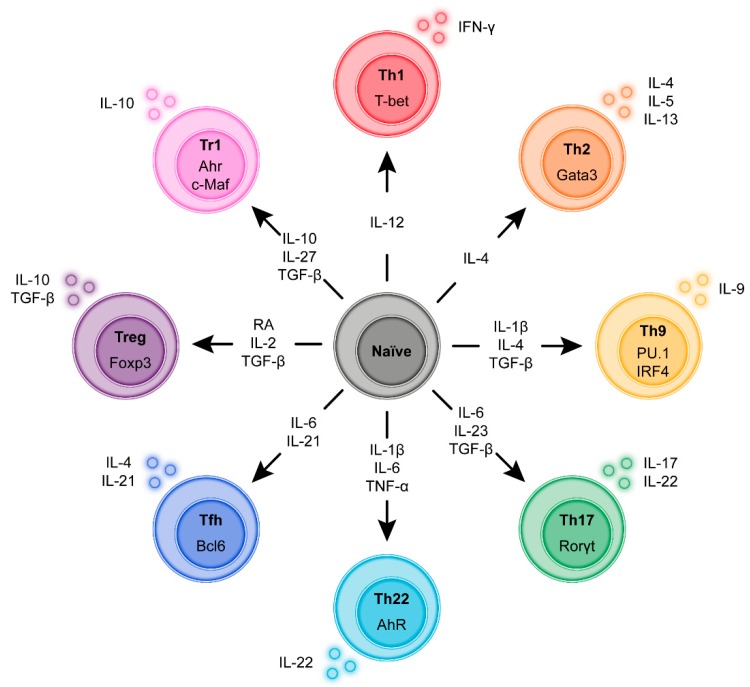
Induction and transcriptional program of the main regulatory and Inflammatory CD4+ T-cell sub-populations. During antigen-specific activation via the T-cell receptor (TCR) and subsequent proliferation in the lymph node naïve CD4^+^ T-cells undergo a functional programming termed differentiation. Depending on the strength of the TCR signal and in response to cytokine co-stimulation by the APC the T-cells express specific transcription factors that skew differentiation to a range of functionalities such as host defense against intracellular pathogens, defense against extracellular pathogens, defense against helminths or immune suppression. Although these differentiation programs are not irreversible classification according to these “subsets” based on dominant cytokine profiles and transcription factors is commonly used to better describe and characterize immune responses.

During antigen-specific activation via the T-cell receptor (TCR) and subsequent proliferation in the lymph node naïve CD4^+^ T-cells undergo a functional programming termed differentiation. Depending on the strength of the TCR signal and in response to cytokine co-stimulation by the APC, T-cells express specific transcription factors that skew differentiation to a range of functionalities such as host defense against intracellular pathogens, defense against extracellular pathogens, defense against helminths or immune suppression. Although these differentiation programs are not irreversible, classification according to these “subsets” based on dominant cytokine profiles is commonly used to better describe and characterize immune responses. In 1986 it was first proposed to classify CD4^+^ T-cells into T helper 1 (Th1) cells and Th2 cells on the basis of their cytokine production [43]. Today, we discern at least seven T helper cell subsets: Th1, Th2, Th9, Th17, Th22, T follicular helper (Tfh) and several types of T-regulatory (Treg) cells (Figure 2) [44,45,46]. For most T-cell subsets, key transcription factors have been identified. Additionally, T-cell subsets are characterized by the expression of distinct members of the signaling transducer and activator (STAT) family. Moreover, depending on CD4^+^ T-cell activation, polarization, and differentiation, the chemokine receptor profile expressed by T helper cells is adapted, which in part is maintained in memory T-cells after the inflammatory response is resolved [47]. Below, we will briefly introduce these Th subsets.

### 3.1. T Helper 1 (Th1) Cells

Th1 cells are essential for elimination of intracellular pathogens. Th1 cells produce interferon gamma (IFN-γ) and Tumor Necrosis Factor alpha (TNF-α) which activate innate immune cells among which phagocytes such as neutrophils and macrophages and structural tissue cells such as epithelial cells and myofibroblasts [48,49]. Th1 polarization is driven by APC-derived IL-12. IL-12 signaling via STAT4 and induction of the key Th1 transcription factor T-box-containing protein (T-bet), encoded by the gene *TBX21*, drives Th1 differentiation [48,49]. T-bet increases up-regulation of IL-12Rβ2 allowing synergistic IL-12 and STAT4 signaling to further increase IFN-γ production.

### 3.2. T Helper 2 (Th2) Cells

Th2 cells control helminth infections, eliminate extracellular microbes and provide B-cell help leading to IgE responses [50]. Th2 cells produce interleukin (IL)-4, IL-5, and IL-13. Th2 polarization is driven by IL-4 via STAT6 signaling. The transcription factor GATA-3 is the most dominant regulator of Th2 cell differentiation [51,52]. Uncontrolled Th2 responses can lead to allergies and asthma.

### 3.3. T Helper 9 (Th9) Cells

Th9 T-cells are closely related to the Th2 subset and provide protection against helminth infections [53] and play a detrimental role in allergy and autoimmunity [54]. Th9 cells are characterized by IL-9 production but may also secrete anti-inflammatory cytokines such as IL-10 [55,56]. Like Th2 cells, Th9 cells require IL-4 signaling via STAT6 to differentiate but in addition require the presence of transforming growth factor-beta (TGF-β) [55,57]. Multiple transcription factors among which PU.1 and Interferon-regulatory factor 4 (IRF4) are required for Th9 cell differentiation and subsequent IL-9 production [58,59]. Recent new findings suggest that a combination of IL-4 and IL-1β may also drive Th9 polarization [60].

### 3.4. T Helper 17 (Th17) Cells

Th17 cells provide protection against bacteria and fungi at mucosal surfaces but are also important drivers of autoimmune diseases [15]. Th17 cells are induced in response to IL-6, IL-23, and TGF-β [61,62,63,64]. Via STAT3, IL-6 induces expression of retinoic acid receptor-related orphan nuclear receptor gamma (RORγt), leading to production of Th17 cytokines IL-17 and IL-22 [65,66]. Pro-inflammatory IL-17 mediates the induction of neutrophil-specific chemokines (CXCL1, CXCL2, CXCL5, CXCL8), induces granulopoiesis factors (granulocyte colony-stimulating factor) and mediators of the acute phase response including IL-6 and stimulates matrix metalloproteinases. However, counterintuitively IL-17 and IL-22 responses elicited by commensals can also support intestinal homeostasis by producing barrier-protective cytokines and antimicrobial peptides [67,68]. As such, while the Th17-associated IL-23-IL-17 axis is thought to play a role in many autoimmune and chronic inflammatory diseases [69], Th17 cells can cooperate with Treg to promote the repair of damaged epithelial barrier during colitis [68,70].

### 3.5. T Helper 22 (Th22) Cells

Th22 cells produce the IL-10 family member IL-22, but not IFN-γ or IL-17. IL-22 is known to protect against tissue damage and bacterial infection [71,72,73]. Th22 cells can express the chemokine receptors CCR10, CCR6, and CCR4. Differentiation of Th22 cells requires the presence of IL-6, TNF-α, and IL-1β, and is suppressed by TGF-β [74,75]. Although Th22 cells do not produce IFN-γ, differentiation of Th22 cells depends on T-bet as well as expression of the aryl hydrocarbon receptor (AhR) [71].

### 3.6. T Follicular Helper (Tfh) Cells

Tfh cells are involved in providing help for B-cell class-switching for immunoglobulin production and germinal center formation [76] in lymphoid tissue. Tfh cells are characterized by expression of the transcription factor BCL-6 [77,78,79] and the chemokine receptor CXCR5 allowing migration along the CXCL13 chemokine gradient present in the B-cell zone [80]. Tfh cell differentiation requires activation of the inducible costimulator (ICOS) and IL-21, IL-6 and STAT3 signaling [81]. Depending on the inflammatory environment, Tfh cells can acquire low to intermediate levels of T-bet, GATA-3, or RORγt which results in various degrees of stimulation of B-cell class-switching [82,83]. Forkhead box P3 (Foxp3) expressing Tfh cells denoted as T follicular regulatory (Tfr) cells function as regulators of the germinal center reaction [84]. As there is very limited knowledge of Tfh cells in IBD these cells will not be discussed in further detail.

### 3.7. T-Regulatory (Treg) Cells

Tregs cells that are stably expressing the lineage-defining transcription factor Foxp3 suppress inflammation through inhibition of other T-cells and by regulating immune cells in their environment. Tregs are classified into thymic-derived Tregs (tTregs) peripherally derived Tregs (pTreg), both of which suppress immune responses and maintain peripheral tolerance [85,86,87,88]. Thymus-derived Treg arise during CD4^+^ T-cell development in the thymus under the influence of relatively high avidity interactions of the TCR with self-antigens [89]. Conversely, pTreg differentiate from naïve CD4^+^ T-cells after activation with exogenous antigen under tolerogenic conditions in secondary lymphoid tissues, in particular in intestinal draining lymph nodes [90,91,92]. Differentiation of peripheral Tregs requires high concentrations of TGF-β and sustained high levels of Foxp3 expression [93,94,95]. Lower or transient levels of Foxp3 expression do not give rise to suppressive Treg and are detected in human non-regulatory recently activated T-cells [96]. Besides TGF-β, additional factors such as vitamin-A derived retinoic acid increase pTreg differentiation [97,98,99]. It is now widely accepted that Tregs are abundant in the intestine and are crucial to antagonize inflammatory CD4^+^ T-cells [100,101]. Tregs suppress through multiple mechanisms, including IL-2 scavenging, the production of regulatory cytokines such as interleukin 10 (IL-10) [102], IL-35 [103] and TGF-β [104,105], and high expression of co-inhibitory receptors such as cytotoxic lymphocyte antigen 4 (CTLA-4) and programmed cell death-1 (PD-1) [106,107].

### 3.8. Type 1 Regulatory T (Tr1) Cells

Tr1 cells are a distinctive population of immunosuppressive anergic T-cells that produce the regulatory cytokines interleukin-10 (IL-10) and TGF-β but do not constitutively express Foxp3 [108,109]. Whether Tr1 cells form a separate lineage is still under discussion as they can both differentiate from naïve T-cells as well as memory T-cells [109]. Tr1 cell differentiation from naïve T-cells can be induced with IL-10 (human and mouse) and IL-27 (mouse). Intriguingly, in agreement with the possible conversion of tissue memory cells into Tr1 cells, the extracellular matrix component Hyaluronic acid promotes the differentiation of Tr1 cells from CD4^+^ memory T-cells [110]. As Tr1 cells can arise at the stage of naïve or memory cell differentiation upon different cues, multiple, possibly redundant transcription pathways are implicated in their differentiation [109].

The function of Tr1 cells in the intestine has mostly been studied in mouse models. Tr1 cells can inhibit inflammatory T-cell responses and colitis by secreting IL-10 and TGF-β [111,112,113,114]. Tr1 cells appear to have distinct functions from IL-10 secreting Treg cells which may depend on their location in the gastrointestinal tract. In the small intestine, Tr1-like CD4^+^Foxp3^neg^ T-cells are the predominant source of IL-10, while in the colonic lamina propria nearly all IL-10 producing cells are Foxp3^+^ Tregs [92,115,116,117]. The essential role of IL-10 in protecting from intestinal inflammation is undisputed as both humans and mice with defects in IL-10 signaling develop severe colitis that is dependent on microbial colonization [118,119,120,121].

### 3.9. T-Cell Plasticity

At this point it is of key importance to realize that these T helper cell subsets can have intermediate stages and have considerable plasticity. For example, Th17 cells can be reprogrammed adopting features of Th1 cells or Tr1-like cells. Such plasticity depends on the tissue environment and cellular cues. Also, human studies demonstrate that specific inflammatory cytokine microenvironments in tissue allow for such conversion of one CD4^+^ T-cell effector phenotype to another [122,123].

Especially Th17 and pTreg cell subsets can co-express multiple transcription factors, cytokines and chemokine receptors [44,124]. As such, fully committed Th17 cells can convert to IFN-γ-producing cells through stimulation via IL-12 and IL-23 [125].

Based on several human, murine, and in vitro data, differentiated Th1, Th2 and Th17 cells may be reprogrammed to convert to Tr1 cells [126,127,128]. In mice, IL-27 and TGF-β promote conversion of Th17 cells into Tr1 cells [127,129].

Conversely, Tregs can differentiate into IL-17 producing cells and lose Foxp3 expression in the presence of Th17 cell-inducing cytokines [130]. In addition, in the presence of IL-12, Tregs can acquire Th1 properties, showing reduced suppressive capacity and substantial IFN-γ release while maintaining Foxp3 expression [131]. Increased frequencies of such Th1-like Foxp3^+^ T-cells are detected in blood from relapsing-remitting multiple sclerosis patients and patients with type 1 diabetes [131,132].

Measuring T-cell plasticity in human is challenging and requires in-depth characterization of T-cells in tissue using broad sets of markers. Recent studies using mass cytometry which allows the use of broad antibody panels argue that human CD4^+^ T-cells may have overlapping cytokine profiles and cannot be clearly distinguished into delineated T helper subsets [133]. Although it is still unclear whether T-cell plasticity involves complete epigenetic programming or just a change in the production of multiple cytokines, it has to be considered that CD4^+^ T-cell subsets are not restricted to completely separate lineages and that CD4^+^ T-cells can adapt their phenotype and function while residing in healthy or inflamed tissue.

## 4. T-Cells in IBD–What Are the Most Dominant Responses in Which Patients?

CD4^+^ T-cell infiltration is a key characteristic of chronic intestinal inflammation [10]. In a longitudinal study, using biopsy material from newly diagnosed IBD patients, it was demonstrated that IBD patients showed a distinctive composition of T lymphocyte subsets in their inflamed intestine when compared with healthy individuals [134]. Intestinal tissue of IBD patients with active inflammation had higher percentages of CD3^+^CD4^+^ T-cells compared to intestinal tissue healthy individuals and T-cell maturation status differed, with higher percentages of CD45RO^+^ T-cells [134,135]. Importantly, follow-up specimens of patients with endoscopic inactive disease showed T-cell subset recovery comparable to healthy individuals [134]. Within the total CD4^+^ T-cell population a tightly controlled balance between functional regulatory and inflammatory CD4^+^ T-cell populations is crucial to prevent uncontrolled CD4^+^ T-cell responses and subsequent intestinal tissue damage [136]. While at steady state, T-cells display mainly a regulatory phenotype [137], increased in Th1, Th2 and Th17 responses and reduced Treg and Tr1 responses have all been suggested to play a role in IBD [136,138]. However, in all these studies there is a large interpatient variability in the phenotype of the T-cell response.

Consequently, it is highly desired to differentiate dominant Th responses in individual patients and relate these to disease course and therapy response. How should such complex monitoring be initiated? Of course, it is preferred to study CD4^+^ T-cells in intestinal mucosa where the disease is located. However, when analyzing the phenotype and function of CD4^+^ T-cell populations isolated from human tissue specimens, it is important to realize that focal disease and the difficulty of accurate enumeration are limitations of intestinal biopsy material. Additionally, phenotypic and functional characteristics of CD4^+^ T-cell responses can be monitored in peripheral blood. Analysis of CD4^+^ T-cells in peripheral blood reflects ongoing T-cell priming and differentiation, but a large majority of circulating CD4^+^ T-cells does not home to the intestine. Consequently, monitoring the phenotype of total CD4^+^ T-cells in peripheral blood of IBD patients has not yet yielded consistent changes in regulatory and inflammatory CD4^+^ T-cell populations. However, recent new techniques allow detailed analysis of gut-homing cells only providing a more reliable peripheral blood monitoring and opening up new avenues for monitoring T-cell responses in IBD [26]. Alternative to cellular responses, large scale immune proteomics of peripheral blood may reveal patterns that discriminate subgroups of patients. Below, we describe our current knowledge of CD4^+^ T-cell responses in IBD in the context of the studied patient material (e.g., intestinal biopsies, blood and plasma) and address whether changes in CD4^+^ T-cell responses may possibly be used to stratify subgroups of IBD patients with similar immune pathways involved in disease pathogenesis [55].

## 5. Abundance of IFN-γ Secreting CD4^+^ T-Cells in CD and UC

In agreement with the large heterogeneity in immunopathology within the clinical CD and UC subtypes, there is considerable variability in the amount of IFN-γ release and frequency of IFN-γ producing intestinal CD4^+^ T-cells from patient to patient. In CD patients, a bias towards IFN-γ secreting T-bet^+^CD4^+^ T-cells and low frequencies of IL-5 and IL-13 secreting GATA-3^+^ CD4^+^ T-cells is strongly associated with CD lesional activity in the intestine [139,140]. Moreover, high frequencies of IFN-γ producing cells in intestinal tissue associated with higher Harvey-Bradshaw Index disease activity scores [139]. In contrast, in UC patients, low frequencies of IFN-γ producing cells were associated with high Mayo scores for disease activity [139].

When isolated from the lamina propria, CD patient-derived CD4^+^ T-cells secrete increased amounts of IFN-γ but reduced IL-5 after CD2 and CD28 stimulation when compared to cells from healthy control or UC [140]. In situ, the increased amount of IFN-γ release may both stem from increased production by activated resident memory T-cells as well as higher numbers of infiltrating recently activated IFN-γ secreting cells in the tissue [141]. ELISPOT assays showed increased numbers of IFN-γ secreting CD4^+^ T-cells after CD2 and CD28 stimulation in CD but not UC, particularly when biopsies were obtained from macroscopically inflamed sites [140]. In contrast to the above studies, decreased frequencies of IFN-γ or T-bet^+^ CD4^+^ T-cells in the lamina propria of CD patients compared to healthy individuals have also been reported [139]. Whether this reduced IFN-γ response reflects a different immune pathogenesis in this cohort is unclear. Alternatively, this contradictive finding may reflect a technical variation as enumeration of IFN-γ secreting cells in the lamina propria remains challenging, especially in CD, as CD4^+^ T-cell infiltration in the tissue can be focal and sampling with relatively small specimens may not be representative for responses along the whole tract.

Overall, the strong IFN-γ signature in CD remains apparent when spontaneous IFN-γ release by whole lamina propria tissue specimens containing variable numbers of T-cells are assessed [142]. Also at mRNA level, despite variability in degree of T-cell infiltration, CD patient biopsies display increased *IL12p35* and *IFNG* levels in ileum and colon, respectively [143]. Furthermore, Th1 cell-associated *IL12RB* receptor [144] and transcription factors *STAT4* and T-bet expression increased in lamina propria T-cells in patients with CD [145].

Dominant IFN-γ responses are also detectable in the circulation of CD patients, particularly during active disease [146]. In peripheral blood of CD patients with small intestinal disease, frequencies of CCR9^+^CD4^+^ T-cells are increased approximately five-fold compared to CD patients with isolated colonic disease [147]. As CCR9 is imprinted on these cells during primary differentiation in intestinal draining lymphoid tissue, detection of CCR9 identifies recently differentiated cells that are migrating through the blood to the small intestinal mucosa. Unfortunately, there is little information on the precise cytokine profile of these newly activated effector cells.

In sum, these data argue that dominant IFN-γ Th1-like responses are more strongly associated with CD than UC [148]. Of note, however, is the large variability from patient to patient that is seen in all biological assays. Part of this variability may be explained by temporally regulated cytokine responses with early lesions characterized by a predominant Th1-like signature while later stages may have a Th1/Th17-like signature [149,150]. However, intrinsic differences based on genetic and environmental variation also play a role. It would be of much interest to discern whether subgroups of CD patients with high IFN-γ responses have a different underlying immune dysfunction and subsequent response to targeted treatment compared to patients with a low IFN-γ response. For such a study inclusion of therapy-naïve patients would be preferred as this excludes confounding variation due to therapy intervention.

### Does the Increased IFN-γ Response Contribute to Disease Pathology?

High frequencies of IFN-γ responses by themselves do not prove a pathological role in disease. Evidence for a causative role for IFN-γ in tissue destruction should come from therapeutic intervention and mouse models. Indeed, antibodies specific for IFN-γ are long known to be therapeutically effective in murine transfer colitis models. In contrast, in the human setting, treatment of CD patients with the anti-IFN-γ antibody fontolizumab did not reach the overall expected therapeutic effect on clinical disease score and is often referred to as ineffective. However, from a biological point of view, it is notable that at day 29, endoscopic improvement was observed in patients treated with one intravenous infusion of 4.0 mg/kg fontolizumab compared with placebo [151]. In the small number of patients examined (n = 14), this was associated with reduced intestinal expression of human leukocyte antigen (HLA)-DR, Stat1 and CXCR3 on histology [151]. Other studies confirm that repeated intravenous infusions of neutralizing anti-IFN-γ antibodies doubled clinical response rates with concomitant reduced serum C-reactive protein (CRP) [152,153].

Treatment success of newer drugs such as ustekinumab, a human IgG1 monoclonal antibody that binds specifically to the p40 protein subunit shared by the IL-12 and IL-23 cytokines also argue for an important detrimental role of Th1 cells in CD pathogenesis [154]. The IL-12 and IL23 cytokines, secreted by activated APCs co-stimulate differentiation of naïve CD4^+^ T-cells into Th1 cells and Th17 cells, respectively. However, IL-23 has also been shown to enhance IFN-γ production by patient-derived intestinal lamina propria cells suggesting IL-23 affects Th1 function in CD [155]. Three trials from the Phase III UNITI development program have demonstrated that ustekinumab effectively reduces disease when used as induction or maintenance therapy in patients with CD [156]. More recently, ustekinumab has also demonstrated efficacy for the induction and maintenance of remission in patients with moderate-to-severe UC supporting the concept that the IFN-γ pathway also plays a pathogenic role in UC [157]. It would be of great value to dissect whether inhibition of both IL-12 and IL-23 are equally involved in the beneficial effect of ustekinumab treatment in UC and CD.

## 6. Abundance of IL-17 Secreting CD4^+^ T-Cells in CD and UC

In contrast to the relatively predominant association of IFN-γ with CD when compared to UC, the inflammatory cytokine IL-17 is strongly associated with both CD and UC [158]. In early studies, no distinction was made between IL-17 family members IL-17A and IL-17F. In the inflamed intestinal tissue of active UC and CD patients, T-cells are an important source of this cytokine but IL-17 protein expression is clearly detectable in both CD3^+^ T-cells and myeloid cells [159,160,161]. Within the Th cell population, the number of IL-17 secreting CD4^+^ T-cells is significantly increased in active UC and CD patients compared with inactive patients while undetectable in healthy controls [160]. At mRNA level, IL-17 and IL-23 expression are significantly increased in the mucosa from active UC and CD patients compared to healthy controls [143,159,162,163]. Furthermore, mucosal T-cells from patients with IBD express the Th17 cell-associated surface markers CD161 and IL-23 receptor (IL-23R), and the Th17 cell-associated transcription factors retinoic acid receptor-related orphan receptor-γt (RORγt; encoded by *RORC*), STAT3 and IRF4 [148,164]. There is considerable heterogeneity in the strength of the IL-17 response among patients. In UC, simultaneous mRNA expression of *IL17A*, *IL17F*, *IL21*, *RORC* and *TGFB* were associated with increased Rachmilewitz endoscopic index [160,165].

In CD, elevated fecal IL-17A is detected in active disease, together with increased numbers of IL-23 and IL-17A producing cells within the lamina propria [166]. Serum IL-17 levels are significantly elevated in both CD and UC patients [146,160].

Supporting these in situ observations, spontaneous IL-17 release in in vitro organ culture of biopsies is higher in CD and UC inflamed mucosa than in uninflamed IBD patient mucosa or healthy controls [161]. This was also confirmed to account for IL-17A production [142]. Upon T-cell stimulation with anti-CD3 and CD28 lamina propria mononuclear cells of CD and UC patients produce higher amounts of IL-17 compared to controls [161].

### 6.1. Does the Increased IL-17 Response Contribute to Disease Pathology?

The functional role of IL-17 secreting CD4^+^ T-cells in CD and UC pathogenesis is not straightforward. Both human and mouse studies demonstrate detrimental as well as protective functions of IL-17A in IBD. In mice, IL-23 driven inflammatory IL-17A and IL-6 are essential for T-cell-mediated colitis and blockade of IL-23 with monoclonal antibodies inhibits these responses [167,168,169]. However, neutralization of IL-17 in dextran sulfate sodium colitis (DSS) aggravates disease [170], mice deficient for IL-23p19 are highly susceptible to T-cell dependent trinitrobenzene sulfonic acid (TNBS) colitis [171] and *Il17a* deficient CD45RB^hi^ cells induce a more aggressive wasting disease in T-cell transfer colitis models [172].

This dual role of IL-17 in murine data may have much relevance to human disease as treatment with secukinumab, a human anti-IL-17A monoclonal antibody, had no beneficial effect in patients with moderate-to-severe CD and was associated with higher frequencies of fungal infections which did not occur in larger trials of psoriasis and rheumatoid arthritis [173]. Similarly, brodalumab, a human anti-interleukin-17 receptor monoclonal antibody elicited worsening of disease in patients with moderate-to-severe CD [174]. Together, these results derived from human studies argue that IL-17 exerts important protective functions in CD. The strength of this protective IL-17 function may vary from individual to individual as is inferred by incidental case reports of UC development in secukinumab treated ankylosing spondylitis and psoriasis patients without prior IBD diagnosis [155,175]. Of note, IBD development is rare in the majority of patients treated with secukinumab for these other immune disorders [176]. This individual variation in reaction to IL-17 blockade may relate to genetic background, as genetic polymorphisms in the TL1A gene have been associated with higher sensitivity to adverse effects of IL-17 inhibition in CD [173].

Even though IL-17 inhibitors are not efficacious in CD, selective inhibition of IL-23 is considered a promising therapeutic approach for IBD. Recently, on the basis of the efficacy of ustekinumab IL-12/IL-23 inhibition in CD, and considering possible risks of impeding host immunity and malignancy surveillance during Th1 blockade, newer selective IL-23p19 antagonists among which risankizumab and brazikumab are tried for the treatment of CD [177]. As murine data demonstrate that IL-12 and IL-23 counter regulate each other [171] it will be intriguing to see how these newer antagonists affect intestinal IFN-γ and IL-17 responses.

### 6.2. What Is the Cellular Source of IFN-γ and IL-17: Th1, Th17 or Th1/17?

Although IFN-γ and IL-17 are readily detected in CD4^+^ T-cells in the lamina propria of CD and UC the exact phenotype of the Th cells is most often not assessed. Classification is rudimentary and mostly based on detection of one cytokine in combination with CD4. Intriguingly, more extensive characterizations of intestinal CD4^+^ T-cells revealed cells with mixed IFN-γ and IL-17 profiles (here denoted as IL-17^+^IFN-γ^+^ CD4^+^ T-cells; also named Th1/Th17 cells or Th17.1 cells) in IBD patients but not healthy individuals [161,178,179]. These cells have long been overlooked as the percentages of IL-17^+^IFN- γ^+^CD4^+^ T-cells are lower compared to Th1 and Th17 cells producing one predominant cytokine [161].

Based on murine studies of inflammatory intestinal disease it was suggested that the cellular source of pathogenic IL-17 and IFN-γ in IBD may stem from a such an IL-23 driven IL-17^+^IFN-γ^+^CD4^+^ T-cell response that is absent is healthy tissue [180]. For human material, chemokine receptor expression by CD4^+^ T-cells has proven to be a useful tool to identify such functionally distinct CD4^+^ T-cell sub-populations [178,181,182]. Indeed, a pro-inflammatory Th17 subpopulation with Th17-like (IL-17A, IL-17F, and IL-22) and Th1-like properties (IFN-γ and CXCR3) was identified in CD patients [183]. Despite its Th17 features, this IL-17^+^IFN-γ^+^CD4^+^ subpopulation contained up to 50% of IFN-γ secreting cells, expressed higher levels of IL-23R than Th1, Th2 or Th17 cells and was enriched in expression of the multi-drug resistance type 1 protein (MDR1) [183]. In keeping with murine colitis studies showing that IL-23 promotes inflammation through induction IFN-γ expression in Th17 cells, these MDR^+^ IL-17^+^IFN-γ^+^CD4^+^ T-cells (CCR6^+^CXCR3^hi^CCR4^lo^CCR10^neg^CD161^+^) cells had an inflammatory profile and in addition displayed resistance to glucocorticoids in vitro [180,183].

Together, accumulating evidence from murine and human studies across multiple diseases argue that not all Th17 cells are pathogenic and that deciphering the unique features of the pathogenic sub-populations such as the disease associated IL-17^+^IFN-γ^+^CD4^+^ T-cells holds promise for more targeted therapeutic approaches [184,185,186,187,188,189].

## 7. The Role of IL-4, IL-5, and IL-13 Secreting CD4^+^ T-Cells in IBD

While the hallmark Th2-associated cytokine IL-4 appears to be increased in IBD, several other Th2-associated cytokines including IL-5, IL-6, and IL-13 and their associating transcription factors are implicated in a subgroup of IBD patients. In particular, in UC patients, IL-5 and IL-13 protein secretion by isolated lamina propria CD4^+^ T-cells increased compared to CD4^+^ lamina propria T-cells from CD and healthy controls [140,190,191]. The main source of IL-13 are thought to be NK-T-cells, specialized CD4^+^ T-cells expressing invariant T-cell receptors with specificity for glycolipids rather than protein antigen [190]. In keeping with the heterogeneity of the disease, not all UC patients may exhibit this increased IL-13 signature [142]. Larger UC cohort studies revealed that mRNA levels of IL-5, IL-13, IL-15, and IL-33 are particularly increased in macroscopically inflamed tissue compared to uninflamed tissue and that IL-13 expression in rectal tissue in therapy-naïve patients associates with response to therapy [162,163]. In agreement with the higher IL-13 levels, increased phosphorylation of its downstream target STAT6 is observed intestinal tissue from therapy-naïve UC patients and may correlate with disease severity [192].

In support of this, higher GATA-3 expression, the most dominant transcription factor driving Th2 programming, is seen in colonic tissue from UC patients compared to ileal CD patients and healthy individuals [193,194] and in colonic tissue from UC patients with active disease compared to UC patients with inactive disease [193].

### Does the Increased IL-13 Response Contribute to Disease Pathology?

IL-13 has been reported to deregulate epithelial tight junctions and may promote apoptosis [191]. The excessive production of IL-13 in UC could lead to a toxic effect on colonic epithelial cells and the epithelial barrier. Indeed, in the oxazolone-induced model of colitis, which closely resembles UC, antibody mediated neutralization of IL-13 suppressed intestinal disease [195]. However, IL-13-specific antibodies (anrukinzumab and tralokinumab) have been trialed for therapeutic purposes in UC (tralokinumab as an add-on therapy) but showed limited therapeutic effect [196,197].

Tissue fibrosis is a recognized outcome of IL-13 exposure [198]. Therefore, it has been hypothesized that IL-13 expression is implicated in CD-related intestinal fibrosis. When comparing intestinal tissue samples from fibrotic CD with UC and uninvolved intestinal tissue, IL-13 transcripts were highest in fibrotic CD, but the difference was not statistically significant [199]. Whether IL-13 is involved in CD-related complications such as intestinal stricturing and fistulizing disease, requires further investigations [198,200].

## 8. The Role of IL-9 Secreting CD4^+^ T-Cells in IBD

IL-9 is a pleiotropic cytokine that can be produced by immune and non-immune cells and acts on an equally large spectrum of immune and structural cells. Initially, IL-9 was thought to be associated with Th2 responses, but because of the different transcriptional programming of IL-9 producing CD4^+^ T-cells the cells were denoted as Th9. IL-9 transgenic overexpression in mice elicits increased mast cell numbers in the intestinal mucosa, altered goblet cell function and IL-13 dependent Paneth cell hyperplasia [201]. Both experimental murine colitis models and patient studies argue for a role of CD4^+^ T-cell derived IL-9 in IBD [54]. In particular, in UC, but not CD, expansion of PU.1^+^ lamina propria CD4^+^ T-cells secreting IL-9 are observed [202,203]. The level of IL-9 mRNA expression in intestinal tissue from UC patients correlates with the endoscopic disease score and histological disease activity [202,203]. Higher serum levels of IL-9 also associate with disease activity in UC [204]. In support of this, Th9-like inflammatory profile, IL-9 promoter-binding Smad2 and Smad3 proteins activated by TGF-β and Notch signaling are up-regulated in the inflamed mucosa of UC patients [205].

### Does the Increased IL-9 Response Contribute to Disease Pathology?

The pathological role of IL-9 has mostly been demonstrated in several murine colitis models. Absence of IL-9 protects mice from trinitrobenzene sulfonic acid (TNBS)- induced colitis [206]. Co-transfer of transfer of IL-9 secreting T-cells with CD45RB^hi^ CD4^+^ T-cells aggravates transfer colitis in recombination-activating gene 1-deficient mice [55] and anti-IL-9 antibodies can be used to treat established oxazolone colitis [203]. Whether specific IL-9 blockade in human would yield similar results is unclear. However, JAK inhibitors such as the JAK1-3 inhibitor tofacitinib may in part act through inhibiting the IL-9 pathway since it is one of the multiple cytokines that activates JAK3 [207].

## 9. The Role of IL-22 Secreting CD4^+^ T-Cells in IBD

In healthy tissue, IL-22 is a protective cytokine which promotes mucosal healing. However, uncontrolled IL-22 levels may exacerbate pathology [208]. Precise functioning of IL-22 is ensured via IL-22 binding protein (IL-22BP), a soluble inhibitory receptor which can bind to IL-22 and prevent it from signaling via the IL-22R1 [209]. In lesional tissue of both CD and UC patients, elevated expression of both IL-22 and IL-22BP are detected at mRNA level and immunohistochemistry [210,211,212]. These changes are also reflected by increased IL-22 in the circulation [210,211]. CD4^+^ T-cells are a primary source of the IL-22BP in inflamed intestinal tissue [212,213]. Heterogeneity of these responses between patients may exist and differ with histological damage. For example, CD patients with granuloma are reported to have increased frequencies of IL-22^+^ and IL-22^+^IFN-γ^+^ cells colonic tissue [214].

### Does the IL-22–IL-22BP Pathway Contribute to Disease Pathology?

Based on murine colitis models it is anticipated that high levels of IL22BP promote inflammation by inference of IL-22 mediated mucosal healing [212]. In agreement, upon response to anti-TNF-α therapy intestinal CD4^+^ T-cells isolated from IBD patients exhibit reduced amounts of IL-22BP expression but still express IL-22 [212], and may even up-regulate IL-22 production [215].

## 10. Regulatory T-Cell Populations (Treg and Tr1)

In the healthy intestine CD4^+^Foxp3^+^ Treg cells are abundant and maintain local homeostasis by suppressing unwanted inflammatory responses to harmless antigens from commensals or nutrients. Identification of patients with genetic deficiency in immune regulatory genes have revealed immune pathways that are essential for this process. Such immune deficiency patients with mutations in the *IL10*, *IL10R* and *FOXP3* genes develop severe intestinal therapy resistant inflammation at infant age [118,216,217]. Moreover, a vast number of publications demonstrate that a balanced inflammatory T-cell versus regulatory T-cells response is essential to prevent experimental colitis induction in mice. These findings combined argue that loss of balance between inflammatory T-cells and Treg and Tr1-like T-cells may contribute to chronic inflammation in CD and UC.

Murine studies have shown that Tregs and Tr1-like T-cells exert regulatory function in different intestinal compartments [18,218,219]. In the murine colonic lamina propria, nearly all IL-10 producing cells are Foxp3^+^ Tregs. Conversely, in the murine small intestine, CD4^+^Foxp3^neg^ T-cells are the predominant source of IL-10 [92,115,116,117]. In line with this compartmentalization of intestinal regulatory T-cell subsets, mice that have a specific deletion of IL-10 in Foxp3^+^ Treg develop inflammation specifically in the colon, but not in the small intestine [220]. This illustrates that CD4^+^Foxp3^+^ Tregs and IL-10-producing CD4^+^Foxp3^neg^ T-cells carry out nonredundant functions at different intestinal locations.

Consequently, it has been extremely difficult to enumerate these functionally suppressive T-cell populations in human IBD and to assess whether (1) an overabundance of inflammatory T-cells, (2) a numerical lack of Tregs/Tr1, (3) defective suppressive function of Treg/Tr1 or (4) resistance of inflammatory T-cells to Treg/Tr1 suppression, underlie the imbalance. Moreover, these responses may differ from patient to patient. A key problem is that reliable phenotypic characterization of peripherally induced pTregs and Tr1 cells is complex. Moreover, a functional suppression assay can only firmly establish whether Tregs are suppressive but has technical limitations as it requires many cells.

## 11. The Role of Tregs in IBD

Over the years it has become clear that Foxp3 positivity alone is insufficient to differentiate Tregs because non-regulatory recently activated T-cells can have lower, or transient levels of Foxp3 expression [96]. Besides this, there is transcriptional plasticity and lineage instability allowing for Foxp3 expressing cells to secrete inflammatory cytokines such as IL-17 and IFN-γ [132]. Consequently, measurement using a panel of multiple markers is required to analyze Tregs. Several labs have compared definitions and together propose that combined analyses of CD3, CD4, CD25, CD127, Foxp3, Ki67 and CD45RA allows monitoring of Tregs in clinical samples [221].

As most studies have not used such an extensive characterization a thorough re-investigation is warranted for enumeration of Tregs in intestinal tissue, most ideally in parallel with peripheral blood Tregs enumeration and functional analysis from the same patient. Despite this, the current available data show little evidence to suggest that CD or UC patients simply lack Tregs in the affected tissues. Initial studies reported that the inflamed mucosa contains increased numbers of CD4^+^CD25^hi^ T-cells, Foxp3^+^ T-cells and transcripts for Foxp3 compared to non-inflamed mucosa [222]. Other studies report similar results, and find a proportional increase of CD4^+^ CD25^hi^ Foxp3^+^ T-cells in intestinal tissue of adult and pediatric patients with CD or UC which appears to increase with disease activity [223,224,225,226].

However, more recent data could argue that these Foxp3^+^CD4^+^ T-cells may harbor populations of non-Treg. For example, IBD patients have an increased percentage of Foxp3^+^IL-17^+^CD4^+^ T-cells in the lamina propria [227,228,229,230]. Frequencies of Foxp3^+^IL-17^+^CD4^+^ T-cells are highest in areas of inflamed IBD mucosal tissues [227,228]. The precise origin, stability and pathogenic properties of these double positive CD4^+^ T-cell populations remain to be determined. One possibility is that double positive CD4^+^ T-cells arise from Foxp3^+^ Tregs that acquire the ability to express transcription factors and cytokines associated with inflammatory CD4^+^ T-cells. Alternatively, they could arise from inflammatory CD4^+^ T-cells that acquire Foxp3 expression. In support of the first, analysis of the TCR repertoire revealed that CD derived Foxp3^+^IL-17^+^CD4^+^ T-cells have TCR Vβ region use more similar to Tregs than to Th17 cells [227], suggesting these are Tregs that acquired the ability to secrete IL-17.

In the blood, reduced frequencies of circulating CD4^+^ T-cells expressing Foxp3 have been reported in some IBD studies [222,231,232,233,234,235]. Conversely, frequencies of Foxp3^+^ Tregs have been reported to increase in peripheral blood of IBD patients after anti-TNF-α treatment [236,237,238] but this was not observed in a more recent study [239]. These variable results may stem from the use of different staining panels to enumerate Tregs and may also reflect that monitoring the total circulating CD4^+^ T-cell population is not sensitive enough to detect transient changes in regulatory and inflammatory mucosally imprinted CD4^+^ T-cell responses. Recently, our laboratory has demonstrated that analysis of peripheral blood CD38^+^ effector T-cells (CD4^+^CD38^+^CD62L^neg^), instead of total CD4^+^ T-cells, lowers the threshold for detection of changes in regulatory versus inflammatory gut-homing CD4^+^ T-cell responses. At time of diagnosis in pediatric patients with active IBD, no proportional changes in FoxP3^+^CD127^neg^ T-cells within the circulating CD38^+^ effector T-cell population was detected [26].

Functional in vitro suppression assays argue that CD4^+^CD25^hi^ T-cells isolated from the intestinal mucosa of patients with IBD are suppressive [222,224,225]. However, it is difficult to establish whether such suppressive activity is sustained in the inflammatory environment in situ. Moreover, inflammation may affect the longevity of the cells as CD4^+^Foxp3^+^ T-cells from inflamed colonic tissue appear to undergo apoptosis more readily than CD4^+^Foxp3^+^ T-cells in non-inflamed tissue [233]. Such increased turnover would require replenishment of new cohorts of CD4^+^Foxp3^+^ T-cells that are generated in the MLN and migrate back to the intestinal lamina propria. Recent data suggest that in a subgroup of CD patients peripheral blood CD4^+^CD25^hi^CD127^lo^Foxp3^+^ Tregs express reduced levels of the adhesion molecule α4β7 which may hamper their migratory function and perturb the replenishment of intestinal Treg [240].

### Does Adoptive Transfer of Treg Attenuate Intestinal Disease?

In mouse models, adoptive transfer of pTregs prevents T-cell driven colitis. As the TCR specificity of Tregs can be manipulated, it could be adapted to react to defined antigens relevant for IBD. tTreg infusion has been tried for safety in human disease and yielded promising results in human graft versus host disease [241,242]. However, since it is anticipated that exogenous bacterial proteins drive the T-cell response in IBD, suppression may require pTreg expansion and transfer. Currently, it is unclear what bacterial proteins are recognized by the TCR of pathogenic T-cells in IBD. Therefore, such pTreg treatment strategies require a much deeper understanding of the T-cell response in IBD.

## 12. The Role of Tr1 Cells in IBD

Immunosuppressive Tr1 cells were first identified in a clinical setting of hematopoietic stem cell transplantation (HSCT) [108,243]. Severe combined immunodeficiency syndrome (SCID) patients who developed active tolerance to their HLA mismatched HSCT had developed a high proportion of hypo-responsive host-specific CD4^+^ T-cell clones producing high amounts of IL-10 [108]. Since their identification, defects in Tr1 cells have been implicated in several diseases among which type 1 diabetes, multiple sclerosis, asthma, allergy and psoriasis [109]. Similar to Tregs, a key hurdle for enumerating Tr1 cells is a reliable phenotypic characterization. CD49b and LAG-3 are reported to enrich for human Tr1 cells. In human and mice the frequency of CD4^+^CD45RA^neg^CD49b^+^LAG-3^+^ T-cells strongly enriches for IL-10 producing T-cells that induce immunological tolerance in clinical setting [244,245,246]. It should be noted, however, that none of these surface markers are uniquely expressed by Tr1 cells. For example, also IL-10-producing Foxp3^+^ Treg cells can express high protein levels of LAG-3 [247].

While it is unequivocally established that IL-10 regulation is essential to prevent human IBD [118], very few studies have addressed possible deficits in Tr1 numbers or defects in Tr1 function in human lamina propria. In peripheral blood of CD and UC patients circulating Tr1 frequencies may not differ from those of healthy individuals. Moreover, when isolated the cells have a similar phenotype and cytokine profile [248]. However, human intestinal IFN-γ-producing Tr1 cells co-expressing CCR5 and PD1 were found to down-regulate IL-10 in patients with IBD [249]. As there is much evidence from murine studies that Tr1 cells protect against colitis development further study into these cells and their role in human IBD are pivotal for a better understanding of disease pathogenesis [112,250].

### Does Adoptive Transfer of Tr1 Cells Attenuate Disease?

In view of its clear role in preventing colitis, supplementation of human recombinant IL-10 has been tried as a therapeutic approach for CD. Both subcutaneous delivery [251,252] as well as using genetically modified IL-10 expressing bacteria [253] may show effectiveness, but has not yielded the overall desired clinical results. It is of interest that in moderately active CD patients subcutaneous delivery of 5 µg/kg IL-10 during 28 consecutive days achieved clinical remission and endoscopic remission in 24% of patients suggesting a subgroup of patients did respond to treatment [251]. It is mostly presumed that protein degradation and a suboptimal route of delivery could have hampered therapeutic effects. However, it is also possible that inflammation is associated with down-regulation of the IL-10 receptor on targets cells. Our lab has recently shown that reduced *IL10RA* expression was detected in peripheral blood mononuclear cells of therapy-naïve pediatric IBD patients [254]. In agreement, a proof of concept experiment showed that IL-10 responsiveness is heterogeneous among IBD patients [254]. Patients that were less sensitive to the IL-10 inhibitory effects exhibited clinical and immunological differences at time of diagnosis. Whether these differences reflect a difference in immune pathology, disease severity, or time to diagnosis remain to be elucidated in these future cohort analyses.

Tr1-mediated delivery of IL-10, however, should offer a therapeutic advantage over direct protein delivery because the cells require TCR-mediated activation providing a means to deliver antigen-specific suppression. Additionally, cellular treatment may have a long-lasting effect when the cells remain viable. In a Phase I/II clinical trial with refractory CD, infusion of ovalbumin-specific Tr1 cell clones was tolerated and showed dose-dependent efficacy in patients suffering from severe disease suggesting the approach may have potential [255,256]. In particular, as functionally suppressive Tr1 cells can be isolated from the peripheral blood of IBD patients and expanded in vitro autologous Tr1 cells transfer could be a therapeutic option [248].

## 13. Toward Precision Intervention in Pathological T-Cell Responses in IBD

The increased overall understanding of T-cell function in chronic immune-mediated diseases has led to the development of a broad spectrum of biologic and small molecule therapeutics allowing for precise intervention in inflammatory immune diseases [156,257,258,259,260]. Several of these compounds have already dramatically changed treatment outcomes in IBD [261,262]. In keeping with the heterogeneity of T-cell mediated disease in IBD, robust interventions such as treatment with anti-TNF-α antibodies are not effective in all patients, and patients who initially respond to anti-TNF-α can lose responsiveness over time.

Therefore, the most burning remaining question is who should receive what drug at what time of disease? As these novel treatment options have different mechanisms of action, it is crucial to select patient subgroups and time of disease to define which patient will most likely respond to these targeted therapies. As outlined above, in-depth patient characterization can identify patients with similar immune pathways involved in their disease pathogenesis. To achieve this, future patient studies should use robust sets of immunological assays to predict and monitor therapeutic success at an individual level.
